# Four New Gallate Derivatives from Wine-Processed Corni Fructus and Their Anti-Inflammatory Activities

**DOI:** 10.3390/molecules26071851

**Published:** 2021-03-25

**Authors:** Hong-Bin Li, Qing-Mei Feng, Ling-Xia Zhang, Jing Wang, Jun Chi, Sui-Qing Chen, Zhi-Min Wang, Li-Ping Dai, Er-Ping Xu

**Affiliations:** 1 School of Pharmacy, Henan University of Traditional Chinese Medicine, Zhengzhou 450046, China; lhb0127@163.com (H.-B.L.); fengqingmei1991@163.com (Q.-M.F.); zhanglingxia1205@126.com (L.-X.Z.); wjlucky666@163.com (J.W.); chijun16@126.com (J.C.); suiqingchen0371@163.com (S.-Q.C.); 2 Engineering Technology Research Center for Comprehensive Development and Utilization of Authentic Medicinal Materials in Henan Province, Henan University of Chinese Medicine, Zhengzhou 450046, China; zhmw123@163.com; 3 National Engineering Laboratory for Quality Control Technology of Chinese Herbal Medicines, Institute of Chinese Materia Medica, China Academy of Chinese Medical Sciences, Beijing 100700, China

**Keywords:** wine-processed Corni fructus, gallate derivatives, anti-inflammatory activity

## Abstract

Four new gallate derivatives—ornusgallate A, *ent*-cornusgallate A, cornusgallate B and C (1a, 1b, 2, 3)—were isolated from the wine-processed fruit of *Cornus officinalis*. Among them, 1a and 1b are new natural compounds with novel skeletons. Their chemical structures were elucidated by comprehensive spectroscopy methods including NMR, IR, HRESIMS, UV, ECD spectra and single-crystal X-ray diffraction analysis. The in vitro anti-inflammatory activities of all compounds were assayed in RAW 264.7 cells by assessing LPS-induced NO production. As the result, all compounds exhibited anti-inflammatory activities at attested concentrations. Among the tested compounds, compound 2 exhibited the strongest anti- inflammatory activity.

## 1. Introduction

Corni fructus is the dried ripe fruit of cornaceae plant *Comus officinalis* Sieb. et Zucc, and is used commonly as a medicinal material [1]. It is mainly distributed in provinces of Henan, Zhejiang, and Shaanxi in China. Corni fructus was first recorded in Shen Nong’s Materia Medica [2]. It has the efficacy of astringing yin qi and anti-hidropoiesis, which is mainly used for treating spontaneous perspiration, night sweat, spermatorrhea, and urorrhea. For example, *Cornus Officinalis* powder (*The Peaceful Holy Benevolence Formula*) is administered to treat kidney diseases and polyuria. Wine-processed Corni fructus, as the main processed product from Corni fructus, is produced according to the method of stewing with wine or steaming with wine (General rule 0213). The clean ripe fruit of *C. officinalis* is stewed or steamed until the wine is absorbed completely to obtain wine-processed product [3]. It has the efficacy of nourishing the liver and kidneys as well as inducing astringency and relieving desertion [2,3,4]. In modern phytochemistry research, more than 300 compounds were isolated from Corni fructus, out of which iridoid, flavone, triterpene, organic acid, and penylpropanoid are the major ones [5,6,7,8,9]. Wine-processed Corni fructus is often adopted as a medicine in TCM clinical practice. In addition, wine-processed Corni fructus is also the principal raw material of some Chinese patented medicines, such as Liuwei Dihuang pills, Qiju Dihuang pills, and Jingui Shenqi pills, all of which were used to nourish the liver and kidneys. The drug property and clinical efficacy of Corni fructus change significantly after processing [10]. However, there are very few studies done on the chemical components of wine-processed Corni fructus, and its pharmacodynamic material basis has also not been figured out [10,11,12] Therefore, in the present study systematic research was conducted on the chemical components of wine-processed Corni fructus.

In the present study, four new gallate derivatives were isolated from wine-processed Corni fructus, and a preliminary study on anti-inflammatory activity was conducted. From the perspective of structure, they were formed through the condensation of gallate and 5-hydroxymethylfurfural derivatives. It could be speculated that these kinds of compounds are the marker compounds, indicating the differences in efficacy between the raw product and processed product of Corni fructus. In recent years, gallate derivatives have always been a hot topic of research presenting extensive biological activities such as anti-inflammatory activity, oxidation resistance, cardiovascular protection, and hypoglycemic effect [13,14,15,16]. Therefore, in-depth studies need to be systematically performed on the biological activity of these four compounds, as well as the compositional change going from raw products to wine-processed products.

## 2. Results and Discussion

### 2.1. Structure Elucidation

**Figure 1:**
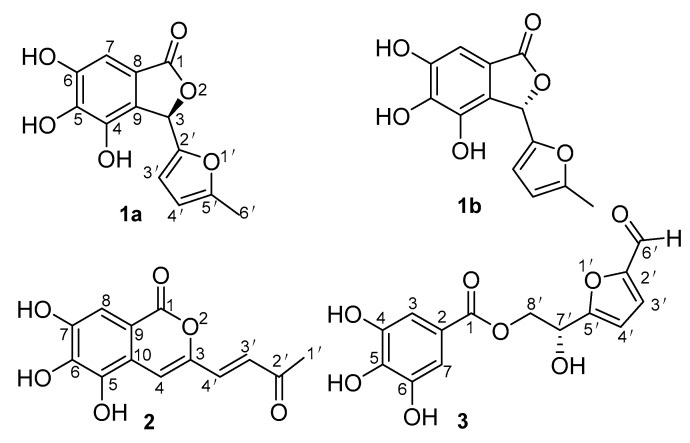
The structures of compounds 1–3.

In order to obtain the 30% ethanol elution fraction, the macroporous resin column chromatography was used to separate the water extract of the wine-processed Corni fructus. The 30% ethanol elution fraction was further isolated by silica gel column chromatography, ODS column chromatography, Sephadex LH-20 chromatography and semipreparative HPLC to obtain four new compounds (Figure 1).

Compounds 1a and 1b were obtained as a mixture, as a yellow columnar single crystal (MeOH). The molecular formula C_13_H_10_O_6_ was deduced from the quasimolecular ion peak at m/z 285.0354 [M + Na]^+^ (calculated for 285.0375, C_13_H_10_O_6_Na) in the HRESIMS with an unsaturation of nine. The UV spectrum of compound 1 showed an absorption maximum at 223, 279 nm. The IR spectrum displayed the presence of hydroxyl (3362 cm^−1^) and carbonyl (1717, 1616 cm^−1^) groups. The ^1^H-NMR data of compound 1 (Table 1) indicated the presence of one methyl group at δ_H_ 2.24 (3H, s, H-6′); three methine protons, including two olefinic methines at δ_H_ 5.98 (1H, d, *J* = 2.6 Hz, H-4′) and 6.22 (1H, d, *J* = 2.8 Hz, H-3′); one oxygenated methine at δ_H_ 6.34 (1H, s, H-3); one aromatic ring hydrogen at δ_H_ 6.85 (1H, s, H-7). ^13^C-NMR data gave 13 carbons, including the following: one methyl, δ_C_ 13.2 (C-6′); one carbonyl carbon, δ_C_ 173.2 (C-1); one oxygenated methine, δ_C_ 75.4 (C-3); four olefinic carbons, δ_C_ 107.3 (C-4′), 112.2 (C-3′), 148.4 (C-2′), and 154.5 (C-5′); a group of aromatic carbons, δ_C_ 103.0 (C-7), 117.5 (C-8), 127.1 (C-9), 141.3 (C-5), 141.4 (C-4), and 149.0 (C-6), as detailed in Table 1. These spectroscopic data revealed that compound 1 is similar to 3-(2-Furyl)-phthalides that has been reported [17].

**Figure 2:**
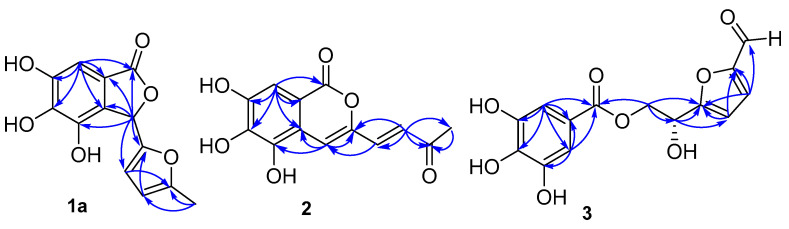
Key HMBC (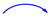) spectra of compounds 1–3.

In combination with analysis of the HMBC spectrum (Figure 2), the NMR data showed the correlations of H-3/C-1, C-2′, C-3′, C-4, C-8 and C-9; H-7/C-1, C-5, C-6, C-8 and C-9; H-3′/C-2′, C-4′ and C-5′; H-4′/C-2′, C-3′, C-5′ and C-6′; H-6′/C-4′ and C-5′. On the basis of detailed analyses of 1D, 2D NMR, the planar structure of compound 1 was determined, as shown in Figure 1.

**Figure 3:**
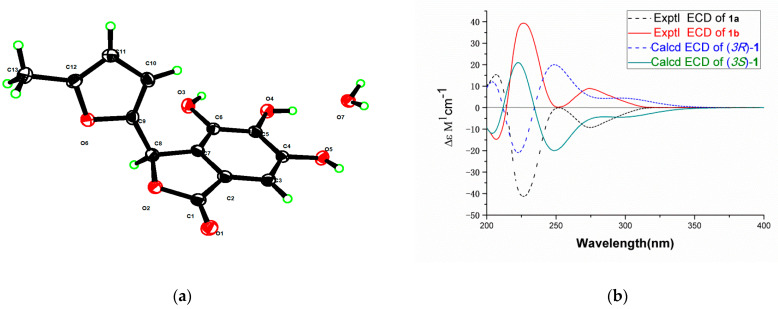
(**a**) The single-crystal spectrum of the enantiomers 1, (**b**) the ECD spectrum of compounds 1a and 1b.

Compound 1, [α] = 0 (c 0.080, MeOH), is a racemix mixture of a pair of enantiomers (1a and 1b), which could undergo rapid interconversion at room temperature. Thus, while performing the chiral separation, their optical rotation and ECD spectra were measured. Compound 1 was treated by normal phase chiral semipreparative column to obtain compounds 1a and 1b. A suitable crystal for X-ray diffraction experiment with Cu-Kα radiation was obtained from MeOH after careful recrystallization to further determine the structure. Compound 1 of CCDC deposition numbers is 2047961. Crystal Data for C_13_H_10_O_6_·H_2_O (*M* = 280.23 g/mol) is as follows: monoclinic, space group P2_1_/n (no. 14), *a* = 10.851 Å, *b* = 4.199 Å, *c* = 26.533 Å, *β* = 92.00, *V* = 1208.2 Å^3^, *Z* = 4, *T* = 170.0 K, μ(CuKα) = 1.094 mm^-1^, *Dcalc* = 1.541 g/cm^3^, 7313 reflections measured (6.666 ≤ 2Θ ≤ 133.512), 2058 unique (*Rint* = 0.0498, *Rsigma* = 0.0436) which were used in all calculations. The final *R1* was 0.0503 (I > 2σ(I)) and *wR2* was 0.1530 (all data). These data can be obtained free of charge via http://www.ccdc.cam.ac.uk/conts/retrieving.html (accessed on 19 January 2021) (or from the CCDC, 12 Union Road, Cambridge CB2 1EZ, UK; Fax: +44 1223 336033; E-mail: deposit@ccdc.cam.ac.uk). The single-crystal spectrum of the enantiomers was determined, as shown in Figure 3, the single-crystal data of Compound 1 is shown in Supplementary Materials Table S1.

To determine the absolute configuration of compounds 1a and 1b, the theoretical ECD was calculated. The ECD spectrum of 1a showed a negative Cotton effect at 225 nm and 275 nm and showed a positive Cotton effect at 207 nm. The ECD spectrum of 1b showed a positive Cotton effect at 225 nm and 275 nm and showed a negative Cotton effect at 207 nm. The calculated curve was in good agreement with that of the experimental one, although a slight peak shift was observed (Figure 3), which indicated that the absolute configuration of 1a was 3*R*, and the absolute configuration of 1b was 3*S*. The structure of 1a and 1b was established as cornusgallate A and *ent*-cornusgallate A. The NMR of racemix mixture of 1a and 1b is shown in Supplementary Materials Figures S4–S8.

Compound 2 was obtained as an amorphous yellow solid. The molecular formula C_13_H_10_O_6_ was deduced from the quasimolecular ion peak at m/z 285.0348 [M + Na]^+^ (calculated for 285.0477, C_13_H_10_O_6_Na) in the HRESIMS with an unsaturation of nine. The UV spectrum of compound 2 showed an absorption maximum at 202, 283, 386 nm. The IR spectrum displayed the presence of hydroxyl (3217 cm^−1^) and carbonyl (1707, 1597 cm^−1^) groups. The ^1^H-NMR data of compound 2 (Table 1) indicated the presence of one methyl group at δ_H_ 2.36 (3H, s, H-1′) as well as three olefinic methines at δ_H_ 6.70 (1H, d, *J* = 15.8 Hz, H-3′), 7.19 (1H, s, H-4) and 7.26 (1H, d, *J* = 12.1 Hz, H-4′). There was one aromatic ring hydrogen at δ_H_ 7.25 (1H, s, H-8). ^13^C-NMR data gave 13 carbons, including the following: one methyl, δ_C_ 27.5 (C-1′); two carbonyl carbons, δ_C_ 163.3 (C-1), and 200.3 (C-2′); four olefinic carbons, δ_C_ 109.6 (C-4), 126.9 (C-3′), 136.0 (C-4′), and 147.8 (C-3); a group of aromatic carbons, δ_C_ 107.2 (C-8), 114.0 (C-10), 120.6 (C-9), 141.3 (C-6), 142.4 (C-5) and 149.5 (C-7), as detailed in Table 1. The aforementioned information indicated that compound 2 could be a gallic acid derivative, which is similar to (E)-5,8-dihydroxy-3-(1-pentenyl)-isocoumarin that has been reported [18].

In the HMBC spectrum (Figure 2), the NMR data showed the correlations of H-4/C-3, C-5, C-10 and C-4′; H-8/C-1, C-6, C-7, C-9 and C-10; H-1′/C-2′ and C-3′; H-3′/C-4/C-1′/C-2′ and C-4′; H-4′/C-3/C-4/C-2′and C-3′. On the basis of detailed analyses of 1D, 2D NMR, the structure of compound 2 was established as cornusgallate B. The NMR of compound 2 is shown in Supplementary Materials Figures S13–S17.

Compound 3 was obtained as an amorphous yellow solid. The molecular formula C_14_H_12_O_8_ was deduced from the quasimolecular ion peak at m/z 331.0441 [M + Na]^+^ (calculated for 331.0430, C_14_H_12_O_8_Na) in the HRESIMS with an unsaturation of nine. The UV spectrum of compound 3 showed an absorption maximum at 201, 218, 280 nm. The IR spectrum displayed the presence of hydroxyl (3347 cm^−1^) and carbonyl (1667 cm^−1^) groups. The ^1^H-NMR data of compound 3 (Table 1) indicated the presence of three methine protons, including two olefinic methines at δ_H_ 6.70 (1H, d, *J* = 3.5 Hz, H-4′) and 7.52 (1H, d, *J* = 3.7 Hz, H-3′); one oxygenated methine at δ_H_ 4.97 (1H, t, *J* = 5.9, 5.8 Hz, H-7′); two aromatic ring hydrogens at δ_H_ 6.92 (2H, s, H-3 and H-7). ^13^C-NMR data gave 12 carbons, including the following: two carbonyl carbons, δ_C_ 165.6 (C-1) and 178.2 (C-6′), four olefinic carbons, δ_C_ 109.8 (C-4′), 124.3 (C-3′), 151.8 (C-2′), 161.4 (C-5′); one oxygenated methine, δ_C_ 64.8 (C-7′); one oxygenated methylene, δ_C_ 65.7 (C-8′); a group of aromatic carbons, δ_C_ 108.7 (C-3), 108.7 (C-7), 119.1 (C-2), 138.6 (C-5), 145.5 (C-4), and 145.5 (C-6), as detailed in Table 1. The aforementioned information indicated that compound 3 could be a gallic acid derivative, which is similar to (5′-Formylfuran-2′-ylmethyl)-4-Hy-droxybenzoate that has been reported [19].

In the HMBC spectrum (Figure 2), the NMR data showed the correlations of H-3/C-1, C-2, C-5, C-6 and C-7; H-7/C-1, C-2, C-5 and C-6; H-3′/C-2′, C-4′, C-5′ and C-6′; H-4′/C-2′, C-4′ and C-5′; H-6′/C-2′; H-8′/C-1, C-5′ and 7′. On the basis of detailed analyses of 1D, 2D NMR, the planar structure of compound 3 was determined, as shown in Figure 1.

**Figure 4:**
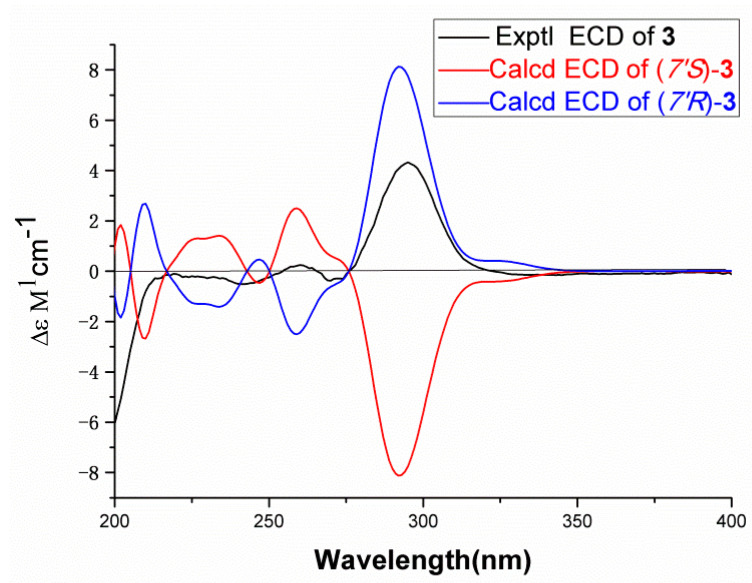
The ECD spectrum of compound 3.

To determine the absolute configuration of compound 3, the theoretical ECD was calculated. The ECD spectrum of compound 3 showed a positive cotton effect at 295 nm (Figure 4), which matched with that of the experimental one perfectly. Thus, the absolute configuration of compound 3 was 7′*R*. The structure of compound 3 was established as cornusgallate C. The NMR of compound 3 is shown in Supplementary Materials Figures S19–S22.

### 2.2. Anti-Inflammatory Effects of Compounds 1–3

The in vitro anti-inflammatory activities of the isolated compounds were assayed in RAW 264.7 cells by assessing LPS-induced NO production [20,21,22,23]. Cell viability assays showed that compounds 1–3 had no cytotoxic activity on RAW 264.7 cells at a concentration of 0–100 μM, and it is not dose dependence (Table S3). To determine if compounds 1–3 can inhibit NO production in LPS-stimulated RAW 264.7 cells, NO concentrations in the culture media containing the compounds were measured using the Griess reaction. Dexamethasone was used as the positive control. As shown in Table 2, compounds 1–3 indicated significant anti-inflammatory activities. Among them, compound 2 has the best anti-inflammatory activity at 50 μM. By comparing the anti-inflammatory activity of compounds 1–3, we found that compounds 1 and 2 have stronger anti-inflammatory activity than 3. The experiment provided a reference for the follow-up study of the anti-inflammatory activity of this plant.

## 3. Discussion

Compounds 1a and 1b are a pair of racemates, which could undergo rapid interconversion at room temperature. The tautomerization mechanism of them may be related to the chemical environment at the allylic position of C-3. The *p*-π conjugation effect and the presence of the ester carbonyl group cause the formation of flat carbocation intermediates, resulting in isomerization. By comparing the anti-inflammatory activity of compounds 1–3, we found that 1 and 2 showed stronger anti-inflammatory activity than 3. The lactone ring may play a role in enhancing the anti-inflammatory activity.

## 4. Materials and Methods

### 4.1. Plant Material

The wine-processed Corni fructus were purchased from Zhengzhou Ruilong Pharmaceutical Co., Ltd., Henan Province, China, and were authenticated by Professor Li-ping Dai, Henan University of Chinese Medicine. A voucher specimen (No. 2018-0413) was deposited at the Engineering Technology Research Center for Comprehensive Development and Utilization of Authentic Medicinal Materials in Henan Province. The processing method of wine-processed Corni fructus is in accordance with the execution standard of the Chinese Pharmacopoeia 2015. Take the clean pulp; add 20–30 kg yellow millet wine for every 100 kg of pulp, and mix well. Moisten it thoroughly, and steam 4–8 h until the wine is exhausted. The surface of the pulp after treatment will appear purple-black or black.

### 4.2. General Experimental Procedures

IR spectra were recorded on a Thermo Nicolet IS 10 spectrometer (Thermo Fisher Scientific, Waltham, MA, USA). UV spectra were recorded on a Thermo EVO 300 spectrometer (Thermo Fisher Scientific, Waltham, MA, USA). Optical rotations were measured using a Rudolph AP-IV polarimeter (Rudolph, Hackettstown, NJ, USA). HR-ESI-MS data was acquired using a Bruker maXis HD mass spectrometer (Bruker, Karlsruhe, Germany). Compounds 1a, 1b, 3 of ECD spectra were measured using an Applied Photophysics Chirascan CD spectropolarimeter (Applied Photophysics, Leatherhead, Surrey, UK), the energy-minimized conformers of 1a, 1b, 3 were generated via the Molecular Mechanics field in Spartan 14, and the geometries were further optimized at the B3LYP/6-31G (d) level in methanol with the integral equation formalism variant polarizable continuum model (IEF-PCM) without vibrational imaginary frequencies. The predominant conformers of compounds 1a, 1b, 3 were subjected to the theoretical calculation of ECD spectra at the RB3LYP/6-31G (d, p) level using the time-dependent density functional theory (TDDFT) method. Compounds 1a, 1b, 3 was drawn via SpecDic software and OriginPro 8 with sigma = 0.16 and UV shift = 10 nm. All the calculations were run with Gaussian 6.1. NMR spectra were recorded on a Bruker Avance III 500 spectrometer (Bruker, Karlsruhe, Germany) with TMS as the internal standard. Chemical shifts (δ) are expressed in ppm with reference to the solvent signals. The enantiomers 1 of X-ray diffractions of all single crystals were carried out at 170.0 K on a Bruker D8 VENTURE (Bruker, Karlsruhe, Germany) diffractometer using Cu-Kα radiation (λ = 1.54178 Å). Integration and scaling of intensity data were performed using the SAINT program. Data were corrected for the effects of absorption using SADABS. The structures were solved by direct method using OLEX2 and SHELXT software [24,25], refined with full-matrix least-squares technique using SHELXL software [26]. Nonhydrogen atoms were refined with anisotropic displacement parameters, and hydrogen atoms were placed in calculated positions and refined with a riding model. Semipreparative HPLC separations were performed on a Qingbohua LC 52 HPLC system, equipped with a dual-wavelength absorbance detector (Qingbohua, Beijing, China). Semipreparative HPLC columns include ODS-AQ (H&E, 10 × 250 mm, 5 μm; H&E Co., Ltd, Beijing, China) and ODS-C18 (ChromCore, 10 × 250 mm, 5 μm, NanoChrom technology (Suzhou) Co., Ltd., Suzhou, China). Chiral-phase separation of the enantiomers 1 was conducted on a Qingbohua LC 52 HPLC system (Qingbohua, Beijing, China), using a COSMOSIL CHiRAL 5C (10ID × 250 mm) (Nacalai Tesque, Inc., Kyoto, Japan). Column chromatography was performed on silica gel (100–200 mesh and 200–300 mesh, Qingdao Marine Chemical Inc., Qingdao, China). TLC was carried out on precoated silica gel GF254 plates. Spots were visualized by heating silica gel plates sprayed with 10% H_2_SO_4_ in ethanol (*v*/*v*).

### 4.3. Cell Lines, Chemicals, and Biochemicals

RAW264.7 macrophages were obtained from the Cell Bank of the Chinese Academy of Sciences (Shanghai, China). LPS, dimethyl sulfoxide (DMSO) and 3-(4,5-dimethyl-2- thiazolyl)-2,5-diphenyl-2-H-tetrazolium bromide (MTT) dye were purchased from Sigma-Aldrich (St. Louis, MO, USA). Dulbecco’s modified eagle medium (DMEM) and Fetal Bovine Serum (FBS) were purchased from Gibco (Carlsbad, CA, USA). The acetonitrile used for HPLC isolation, which was of HPLC grade, was purchased from Fisher (Waltham, MA, USA). The solvents used to column chromatography (Silica gel and Sephadex LH-20 gel column) in the study, such as dichloromethane and methanol, were of ACS grade (Tianjin, China).

### 4.4. Extraction and Isolation

The air-dried fruits (50 kg) of wine-processed Corni fructus were exhaustively extracted with aqueous solution (500 L × 2, 2 h) at reflux. After filtration and evaporation in vacuo, a residue (22.8 kg) was obtained. The residue absorbed on D101 macroporous resin, eluted successively with EtOH/H_2_O (0:100, 30:70, 70:30, *v*/*v*), and eluted in a gradient to obtain three main fractions (Fr. A-Fr. C). The Fr. B (3.2 kg) was taken up in H_2_O, successively extracted with ethyl acetate and *n*-butanol, and recovered by vacuum distillation to obtain ethyl acetate extract, *n*-butanol extract, and H_2_O extract (Fr. B1-Fr. B3). Then, the Fr. B1 (409 g) was further subjected to column chromatography over silica gel (200–300 mesh), and eluted in a step gradient manner with CH_2_Cl_2_/MeOH (100:0 to 0:100, *v*/*v*) to yield eight major fractions (Fr. B1-1-Fr. B1-8). Fr. B1-4 (37.7g) was subjected to ODS column chromatography with MeOH/H_2_O (10:90 to 100:0, *v*/*v*) to obtain four subfractions (Fr. B1-4-1-Fr. B1-4-4). Further separation of Fr. B1-4-3 (4.9 g) by Sephadex LH-20 column chromatography eluted with MeOH to divide into three fractions (Fr. B1-4-3-1-Fr. B1-4-3-3). Fr. B1-4-3-1 was subjected to semipreparative HPLC using 25% CH_3_CN/H2O (3 mL/min, ODS-AQ) to yield compound 1 (200 mg). Compound 1 was subjected to semi-preparative HPLC using 80% *n*-hexane/Isopropanol (3 mL/ min, COSMOSIL CHiRAL 5C) to yield compounds 1a and 1b (95 mg, 95mg). Fr. B1-4-3-3 was subjected to semipreparative HPLC using 20% CH_3_CN/H_2_O (3 mL/min, ODS-AQ) to yield compound 2 (45 mg). Fr. B1-4-2 (4.1 g) by Sephadex LH-20 column chromatography eluted with MeOH to divide into four fractions (Fr. B1-4-2-1-Fr. B1-4-2-4). Fr. B1-4-2-2 was subjected to semipreparative HPLC using 14% CH_3_CN/H_2_O (3 mL/min, ODS-C18) to yield compound 3 (35 mg) (Figure S25).

### 4.5. Compounds Characterization Data

Cornusgallate A (1a): yellow powder, [α] = –367.28 (c 0.019, *n*-hexane/Isopropanol (80:20)); UV (MeOH) λmax (log *ε*): 223 (2.39) nm, 279 (4.12) nm; ECD (*n*-hexane/Isopropanol (80:20)) 207 (Δ*ε* +15.41), 225 (Δ*ε* –40.92), 275 (Δ*ε* –9.22) nm; IR (iTR) νmax: 3362, 2923, 2852, 1717, 1616, 1484, 1300, 1072, 949, 880 cm^–1^; ^1^H-NMR (MeOD-*d*4, 500 MHz) and ^13^C-NMR (MeOD-*d*_4_, 125 MHz) spectral data see Table 1; (+)-HRESIMS m/z 285.0354 [M + Na]^+^ (calculated for C_13_H_10_O_6_Na, 285.0375).

*ent*-cornusgallate A (1b): yellow powder, [α] = +236.20 (c 0.019, *n*-hexane/ Isopropanol (80:20)); UV (MeOH) λmax (log *ε*): 223 (2.39) nm, 279 (4.12) nm; ECD (*n*-hexane/Isopropanol (80:20)) 207 (Δ*ε* –14.81), 225 (Δ*ε* +39.07), 275 (Δ*ε* +8.89) nm; IR (iTR) νmax: 3362, 2923, 2852, 1717, 1616, 1484, 1300, 1072, 949, 880 cm^–1^; ^1^H-NMR (MeOD-*d*4, 500 MHz) and ^13^C-NMR (MeOD-*d*_4_, 125 MHz) spectral data see Table 1; (+)-HRESIMS m/z 285.0354 [M + Na]^+^ (calculated for C_13_H_10_O_6_Na, 285.0375).

Cornusgallate B (2): yellow amorphous powder, UV (MeOH) λmax (log *ε*): 202 (3.52), 283 (2.60), 386 (3.13); IR (iTR) νmax: 3217, 2924, 1707, 1597, 1369, 1076, 970, 864 cm^–1^; ^1^H-NMR (MeOD-*d*_4_, 500 MHz) and ^13^C-NMR (MeOD-*d*_4_, 125 MHz) spectral data see Table 1; (+)-HRESIMS m/z 285.0348 [M + Na]^+^ (calculated for C_13_H_10_O_6_Na, 285.0375).

Cornusgallate C (3): yellow powder, [α] = +41.33 (c 0.066, MeOH); UV (MeOH) λmax (log *ε*): 201 (3.83), 218 (3.55), 280 (3.65); ECD (MeOH) 295 (Δ*ε* +4.33) nm; IR (iTR) νmax: 3347, 2922, 2851, 1667, 1451, 1215, 1038, 874, 769 cm^–1^; ^1^H-NMR (DMSO-*d*_6_, 500 MHz) and ^13^C-NMR (DMSO-*d*_6_, 125 MHz) spectral data see Table 1; (+)-HRESIMS m/z 331.0441 [M + Na]^+^ (calculated for C_14_H_12_O_8_Na, 331.0430).

### 4.6. Anti-Inflammatory Bioassays

Cell culture: RAW264.7 cells were cultured in DMEM complete medium supplemented with 10% neonatal bovine serum. Cells were maintained at 37 °C under a humidified atmosphere of 5% CO_2_ in an incubator.

Cell viability assay: Cell viability was evaluated by MTT reduction assay. Briefly, RAW 264.7 cells were seeded on 96-well microtiter plates at 1.0 × 10^5^ cells/well for 24 h and treated with each compound (0, 6.25, 12.5, 25, 50, and 100 µM). After being treated with tested samples for 24 h, the medium was removed and the cells were incubated with MTT (1.0 mg/mL, 10 µL for 3 h at 37 °C. The formazan crystals in the cells were dissolved in DMSO. The levels of MTT formazan were measured as absorbance at 490 nm. The cell survival rate was calculated [20,21,22,23].

Measurement of NO release and NO inhibition rate: Accumulation of nitrite, an indicator of NO synthase activity, in culture medium was measured using the Griess reaction. Cells (2 × 10^5^ cells/well) were cultured on 24-well microtiter plates for 12 h, then treated with each compound (20, 50 µM) for 1 h. LPS (1 µg/mL) was added to the medium and cultured for 24 h. Fifty microliter culture medium supernatants were mixed with 50 µL Griess reagent (part I: 1% sulfanilamide; part II: 0.1% naphthyl ethylene diamide dihydrochloride and 2% phosphoric acid) at 37 °C. After 10 min, the absorbance was measured at 540 nm. Inhibition rate of NO was calculated [20,21,22,23].

Statistical Analysis: All the results were expressed as mean ± standard deviation. Statistical analyses were performed using GraphPad Prism 6.0 software (GraphPad Software, San Diego, CA, USA). Dunnett’s multiple comparison test was employed to perform the one-way analysis of variance to identify the significance of differences among groups. *p* < 0.05 was considered to be statistically significant.

## 5. Conclusions

Four new gallate derivatives were isolated and identified from the wine-processed Corni fructus. The anti-inflammatory activities of these compounds were evaluated against RAW264.7 cells by MTT method, and all the compounds showed anti-inflammatory effects. The skeleton of compounds 1a and 1b was isolated from natural products for the first time, and the previous reports showed that the skeleton was chemically synthesized. The water extract of the wine-processed Corni fructus contains a lot of gallic acid and 5-Hydroxymethylfurfural. Compounds 1 and 2 have more significant anti-inflammatory activity than compound 3, which revealed the structure–activity relationship, that the lactone ring may play a role in enhancing the anti-inflammatory activity. Modern studies believe that the efficacy of wine-processed Corni fructus is stronger than that of Corni fructus. Therefore, it is widely used in clinical practice of traditional Chinese medicine. However, the chemical constituents of wine-processed Corni fructus were rarely reported. Our research provided a foundation for the clinical application of wine-processed Corni fructus.

## Figures and Tables

**Table 1:** ^1^H-NMR and ^13^C-NMR data of Compounds 1–3 ^a^.

No.	Compounds 1a and 1b		Compound 2		Compound 3	
	δ_H_ (*J* in Hz)	δ_C_	δ_H_ (*J* in Hz)	δ_C_	δ_H_ (*J* in Hz)	*δ*_C_
1	-	173.2	-	163.3	-	165.6
2					-	119.1
3	6.34, s	75.4	-	147.8	6.92, s	108.7
4	-	141.4	7.19, s	109.6	-	145.5
5	-	141.3	-	142.4	-	138.6
6	-	149.0	-	141.3	-	145.5
7	6.85, s	103.0	-	149.5	6.92, s	108.7
8	-	117.5	7.25, s	107.2		
9	-	127.1	-	120.6		
10			-	114.0		
1′			2.36, s	27.5		
2′	-	148.4	-	200.3	-	151.8
3′	6.22, d (2.8)	112.2	6.70, d (15.8)	126.9	7.52, d (3.7)	124.3
4′	5.98, d (2.6)	107.3	7.26, d (12.1)	136.0	6.70, d (3.5)	109.8
5′	-	154.5			-	161.4
6′	2.24, s	13.2			9.56, s	178.2
7′					4.97, t (5.8)	64.8
8′					4.37, dd (11.3, 6.4)	65.7
					4.41, dd (11.3, 5.3)	

^a 1^H-NMR data (δ) were measured in MeOD-*d*_4_ at 500 MHz and ^13^C-NMR data (δ) were measured in MeOD-*d*_4_ at 125 MHz for compounds 1–2. ^1^H-NMR data (δ) were measured in DMSO-*d*_6_ at 500 MHz and ^13^C-NMR data (δ) were measured in DMSO-*d*_6_ at 125 MHz for compound 3. Coupling contents (*J*) in Hz are given in parentheses. The assignments were based on HSQC, HMBC experiments.

**Table 2:** Anti-inflammatory effects of compounds 1–3 on LPS-Induced RAW264.7 ^a.^

Sample	*c* (μM)	NO Release (μM)	NO Inhibition Rate (%)
Control	-	8.76 ± 0.84	-
Model ^c^	-	14.23 ± 0.84	- **
1	25	7.89 ± 0.87	115.85 ± 2.86 **
	50	4.11 ± 1.02	184.96 ± 1.73 **
2	25	8.63 ± 0.93	102.26 ± 2.48 **
	50	3.33 ± 0.64	199.09 ± 1.98 **
3	25	11.80 ± 0.56	44.38 ± 1.16 **
	50	7.06 ± 0.69	130.98 ± 1.74 **
dexamethasone ^b^	3	11.49 ± 0.94	50.13±2.71

^a^ Values are means ± SD of three experiments, with each data point done in triplicate. ^b^ Dexamethasone was used as the positive control. ^c^ The model group refers to the LPS-induced RAW264.7 cells without drug stimulation. ** *p* < 0.01.

## Data Availability

Data is contained within the article or supplementary material.

## Supplementary Materials

The following are available online. Figure S1–S8 and S10-S24: IR, UV, HR-ESI-MS, NMR (1D and 2D) spectrum of Compounds 1–3; Figure S9: Single-crystal X-ray diffraction analysis spectrum of the enantiomers 1; Figure S25: Purification process diagram of compounds 1–3; Figure S26: HPLC chart of compound 1 chiral resolution; Table S1 is Single-crystal X-ray diffraction analysis data of the enantiomers 1; Table S2: Anti-inflammatory of the different layers of wine-processed Corni fructus on LPS-Induced RAW264.7; Table S3: The cytotoxic activities of compounds 1–3 on the viability RAW264.7 cells.
