# Supplementary material for: Four New Gallate Derivatives from Wine-Processed Corni Fructus and Their Anti-Inflammatory Activities
Source: Molecules. 2021 Mar 25;26(7):1851. doi: 10.3390/molecules26071851 (PMC8037767; doi:10.3390/molecules26071851)
Supplement: Supplementary file 1 [file molecules-26-01851-s001.pdf]

molecules

Supplementary materials

Article

Four New Gallate Derivatives from Wine-Processed Corni Fructus and Their Anti-Inflammatory Activities

Hong-Bin Li [1,2,†], Qing-Mei Feng [1,2,†], Ling-Xia Zhang[1,2], Jing Wang [1,2], Jun Chi [1,2], Sui-Qing Chen [1], Zhi-Min Wang [2,3], Li-Ping Dai [1,2,*] and Er-Ping Xu [1,2,*]

1 Henan University of Traditional Chinese Medicine, Zhengzhou 450046, China; lhb0127@163.com (H.-B.L.); fengqingmei1991@163.com (Q.-M.F.); zhanglingxia1205@126.com (L.-X.Z.); wjlucky666@163.com (J.W.); chijun16@126.com (J.C.); suiqingchen0371@163.com (S.-Q.C.)
2 Engineering Technology Research Center for Comprehensive Development and Utilization of Authentic Medicinal Materials in Henan Province, Henan University of Chinese Medicine, Zhengzhou 450046, China; zhmw123@163.com (Z.-M.W.)
3 National Engineering Laboratory for Quality Control Technology of Chinese Herbal Medicines, Institute of Chinese Materia Medica, China Academy of Chinese Medical Sciences, Beijing 100700, China
* Correspondence: zzdai@163.com (L.-P.D.); xuerping0371@163.com (E.-P.X.); Tel.: +86-18703651652 (L.-P.D.)
† These authors contributed equally to this work.

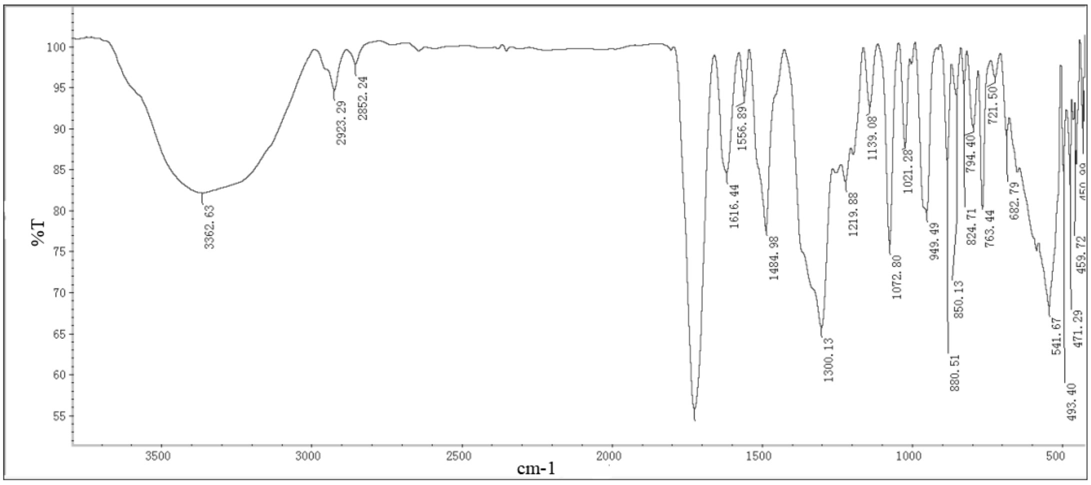

Figure S1. IR spectrum of racemix mixture of **1a** and **1b**.

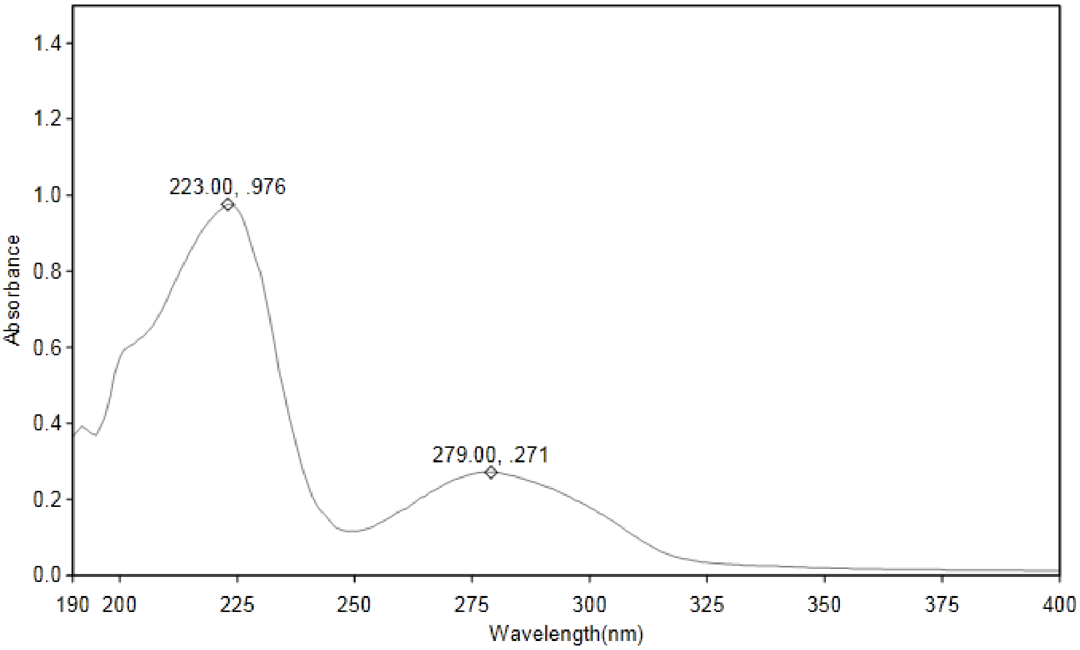

Figure S2. UV spectrum of racemix mixture of **1a** and **1b**.

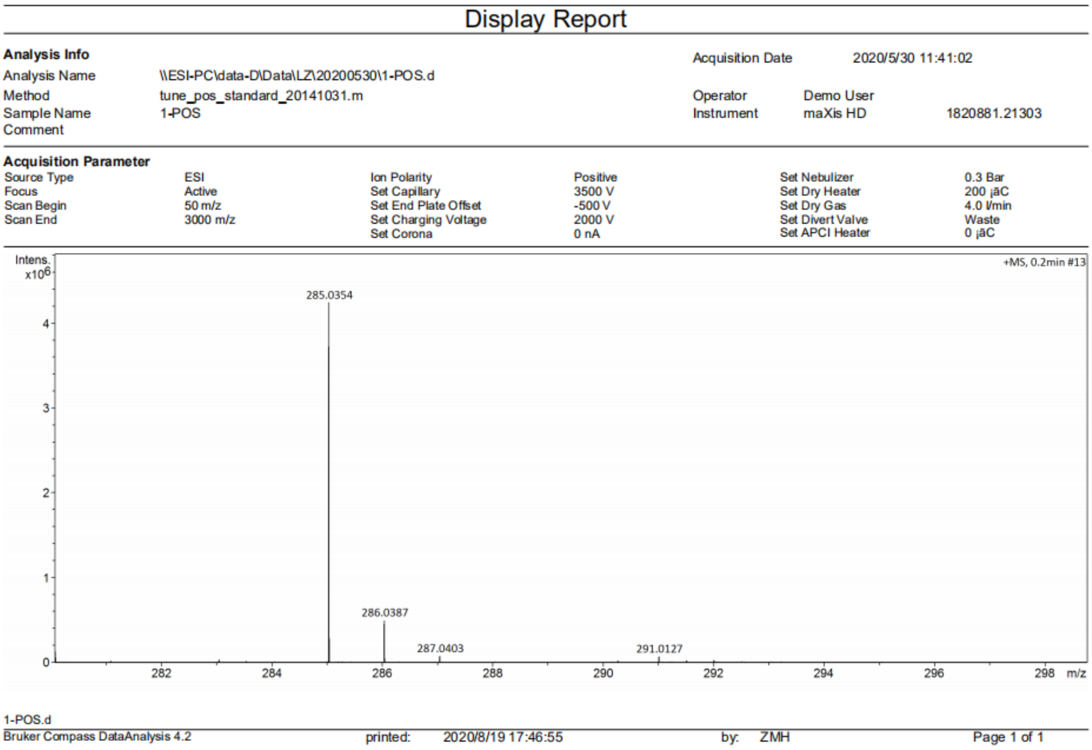

Figure S3. HRESIMS spectrum of racemix mixture of **1a** and **1b**.

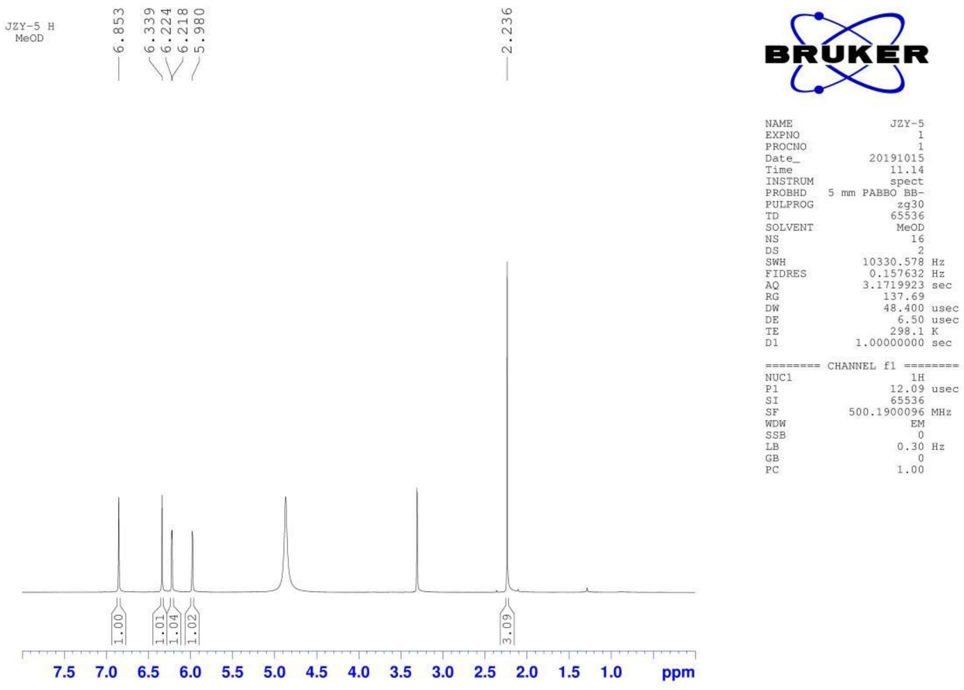

Figure S4. ^{1}H-NMR spectrum of racemix mixture of **1a** and **1b** (MeOD, 500 MHz).

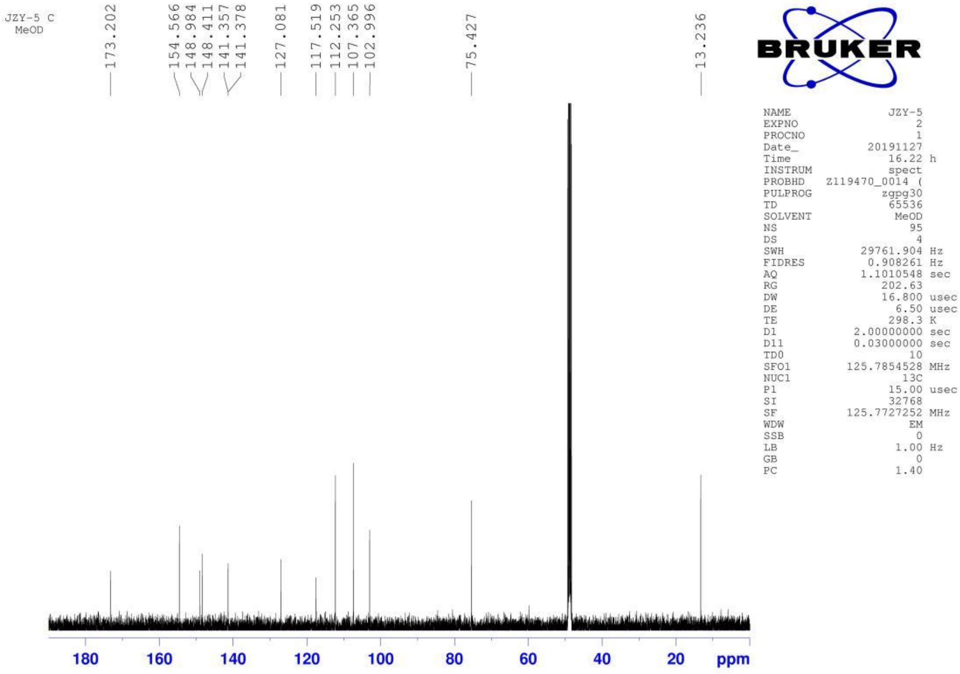

Figure S5. ^{13}C-NMR spectrum of racemix mixture of **1a** and **1b** (MeOD, 125 MHz).

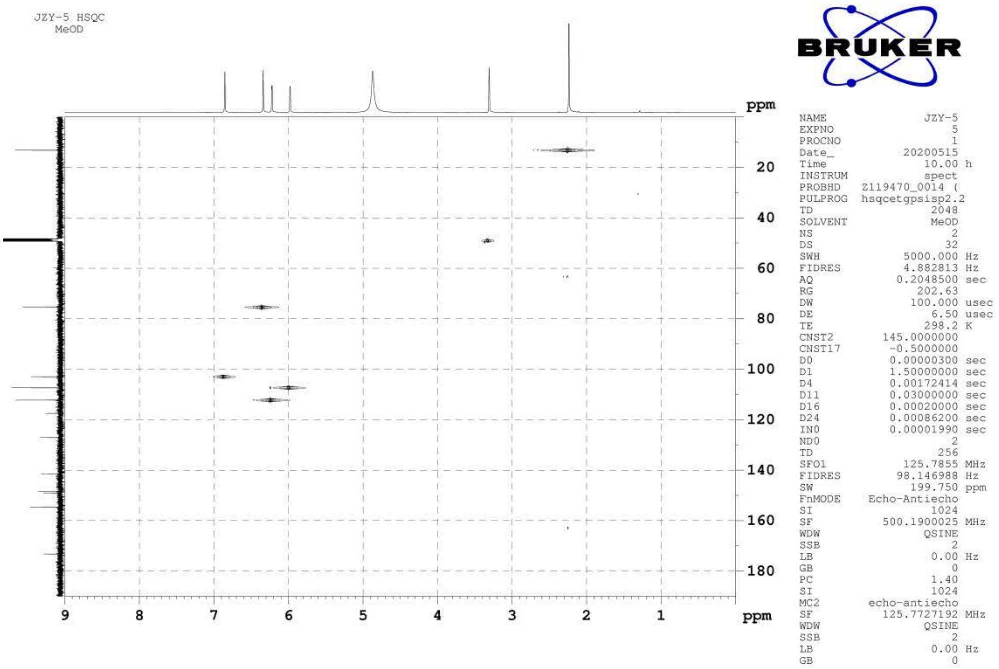

Figure S6. HSQC spectrum of racemix mixture of **1a** and **1b**.

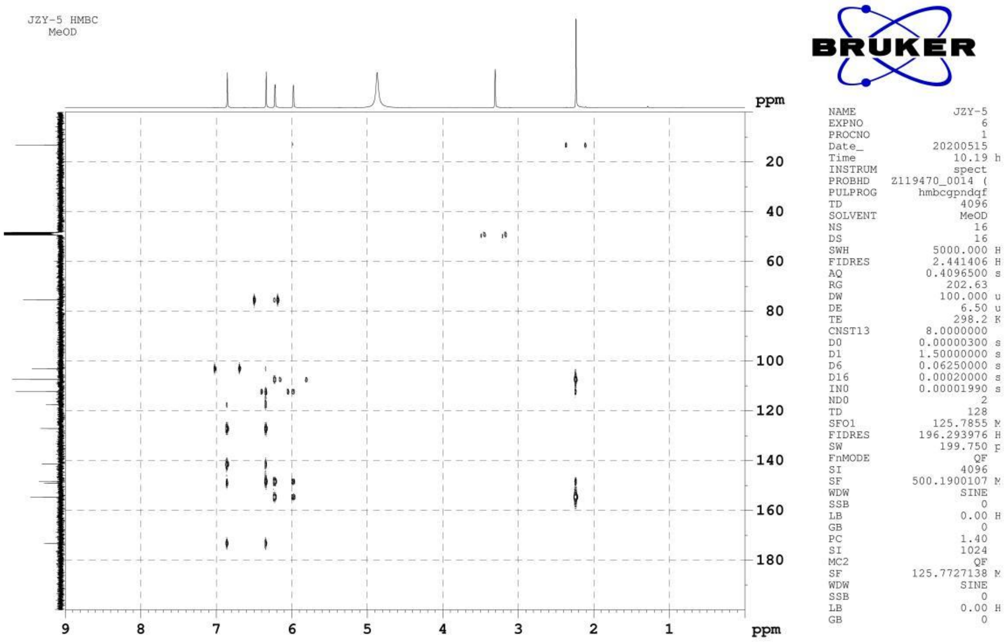

Figure S7. HMBC spectrum of racemix mixture of **1a** and **1b**.

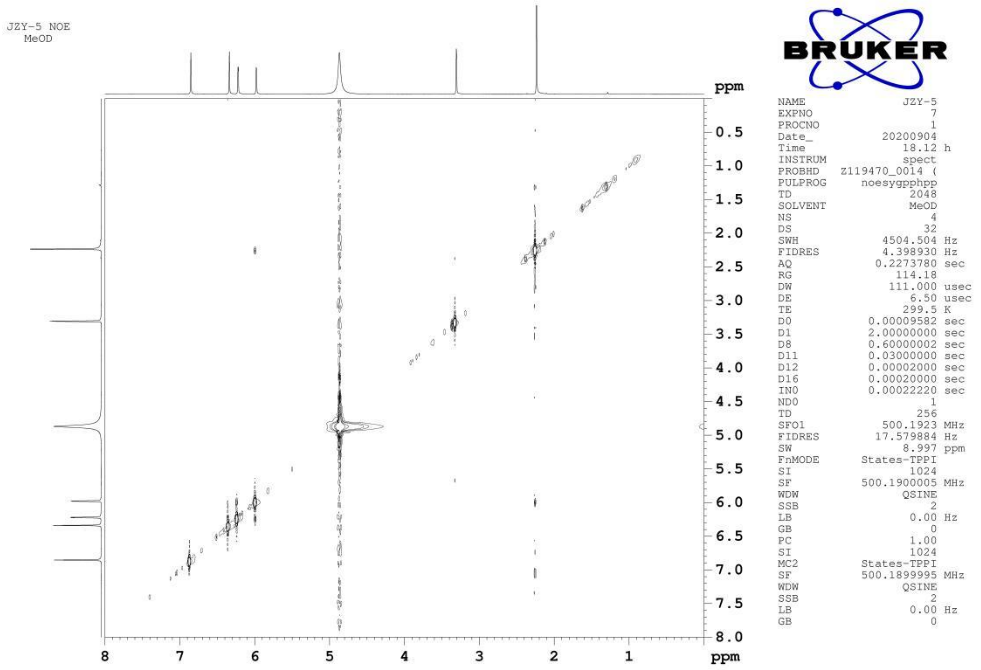

Figure S8. NOESY spectrum of racemix mixture of **1a** and **1b**.

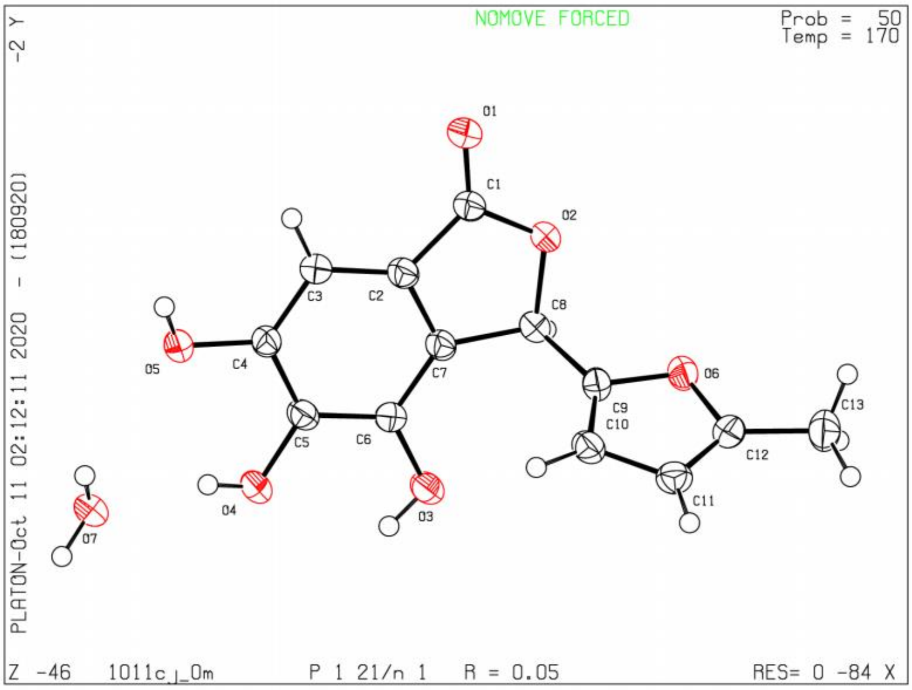

Figure S9. Single-crystal X-ray diffraction analysis spectrum of the enantiomers **1**.

Table S1. Single-crystal X-ray diffraction analysis data of the enantiomers **1** [a].

	Calculated	Reported
Volume	1208.196	1208
Space group	$P2_1/n$	$P12_1/n$
Hall group	– P 2yn	– P 2yn
Moiety formula	$C_{13}H_{10}O_6$, H_2O	$C_{13}H_{10}O_6$, H_2O
Sum formula	$C_{13}H_{12}O_7$	$C_{13}H_{12}O_7$
Mr	280.23	280.23
Dx, g/cm^3	1.541	1.541
z	4	4
Mu (mm^{-1})	1.094	1.094
F000	584.0	584.0
F000′	586.26	
h, k, lmax	12, 5, 31	12, 4, 31
Nref	2110	2058
Tmin, Tmax	0.936, 0.978	0.843, 0.970
Tmin′	0.916	

[a] Bond precision: *C–C* = 0.0037 Å, *Wavelength* = 1.54178; Cell: *a* = 10.85100, *b* = 4.19900, *c* = 26.5300, *alpha* = 90, *beta* = 92.0000, *gamma* = 90; Temperature: 170 K; *Data completeness* = 0.975; *Theta (max)* = 66.756; *R (reflections)* = 0.0503 (1592); *wR2 (reflections)* = 0.1530 (2058); *S* = 1.007; *Npar* = 191.

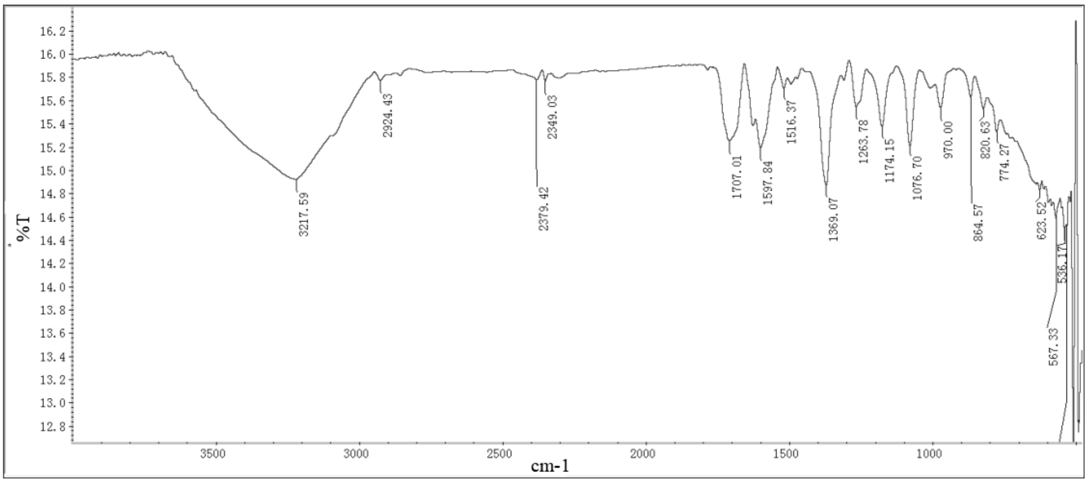

Figure S10. IR spectrum of compound **2**.

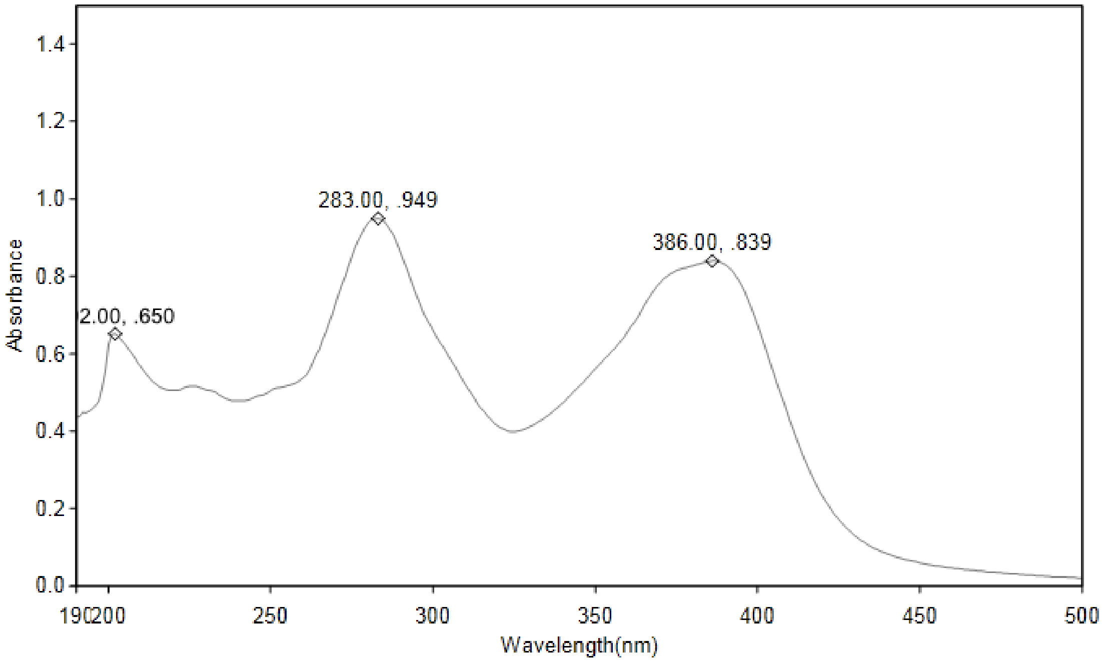

Figure S11. UV spectrum of compound **2**.

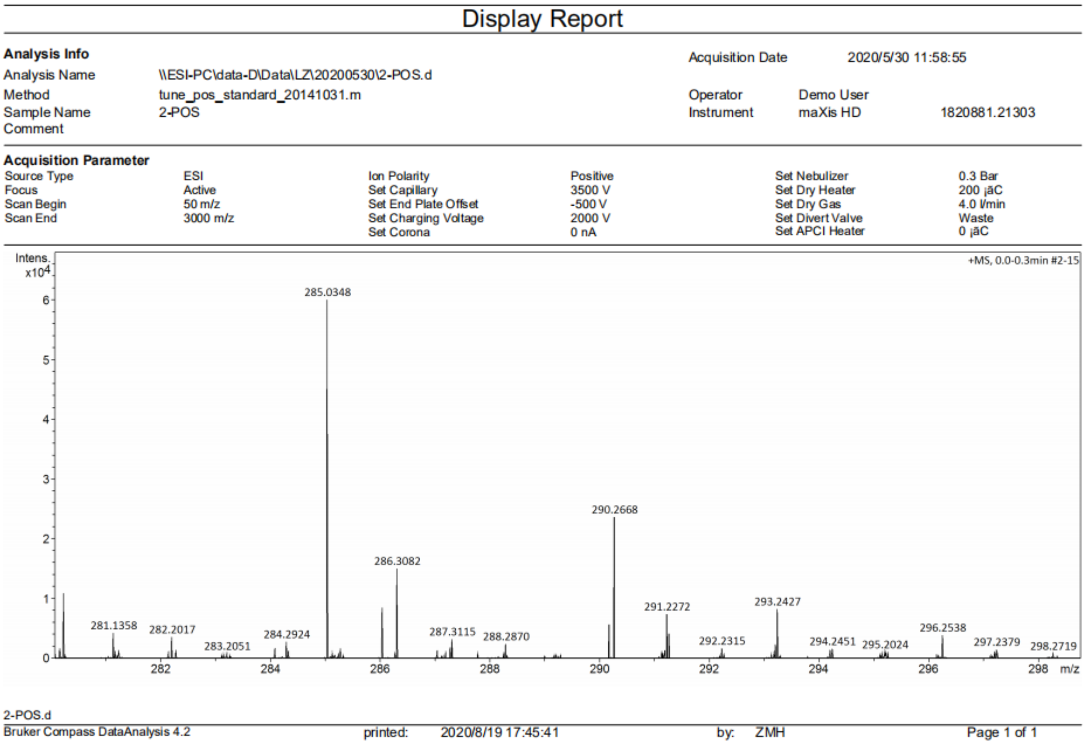

Figure S12. HRESIMS spectrum of compound **2**.

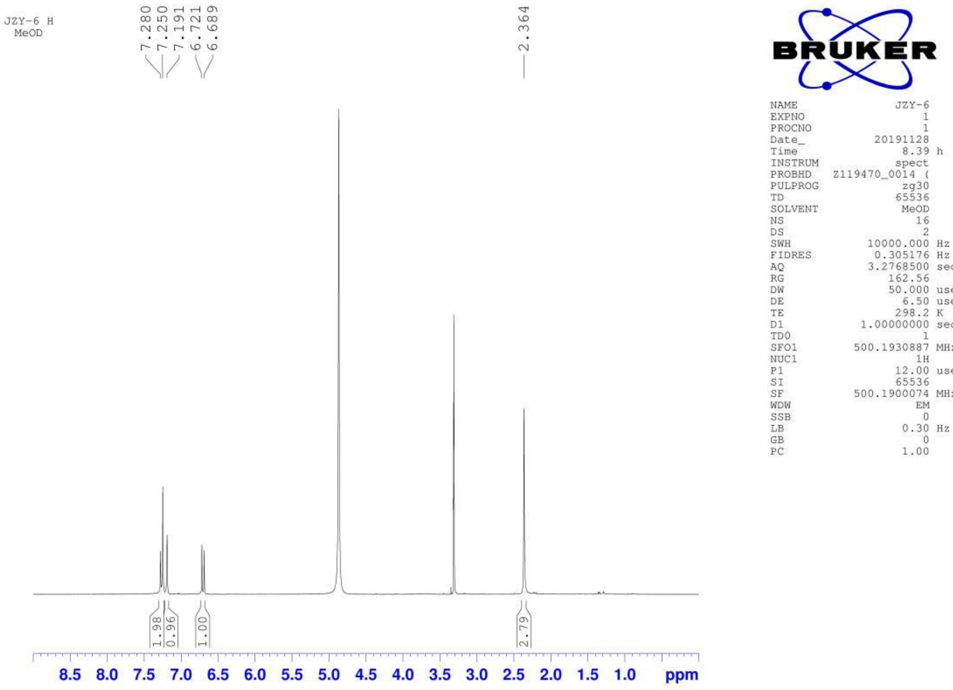

Figure S13. ^{1}H-NMR spectrum of compound **2** (MeOD, 500 MHz).

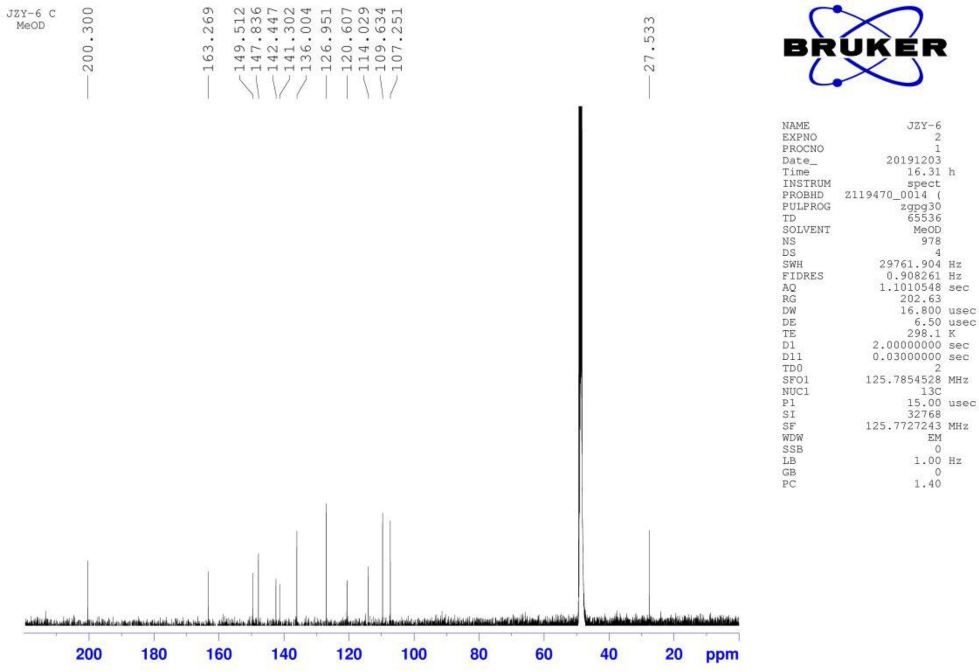

Figure S14. ^{13}C-NMR spectrum of compound **2** (MeOD, 125 MHz).

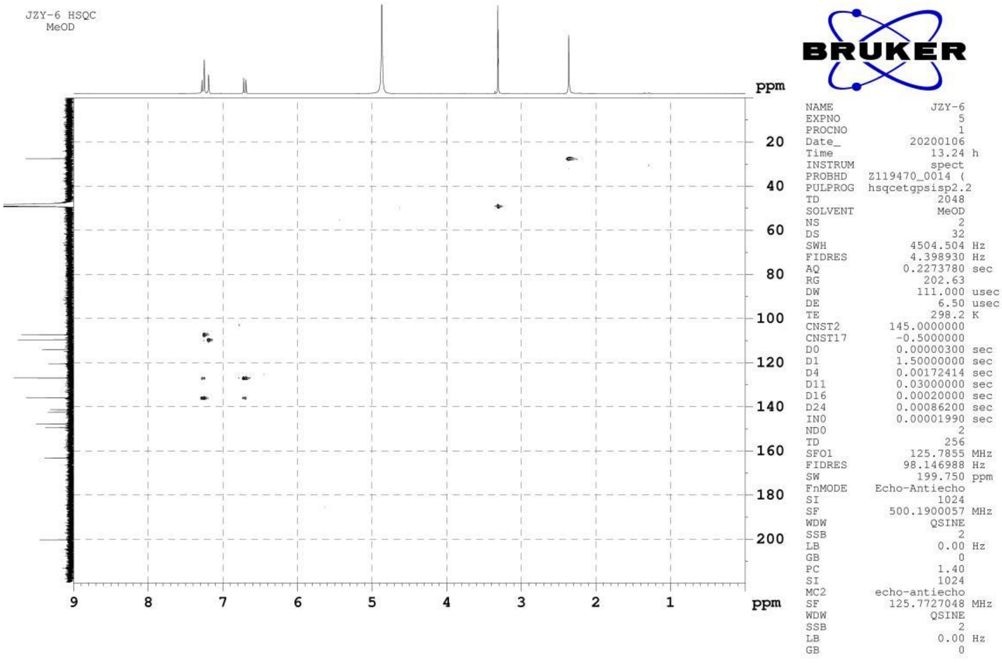

Figure S15. HSQC spectrum of compound **2**.

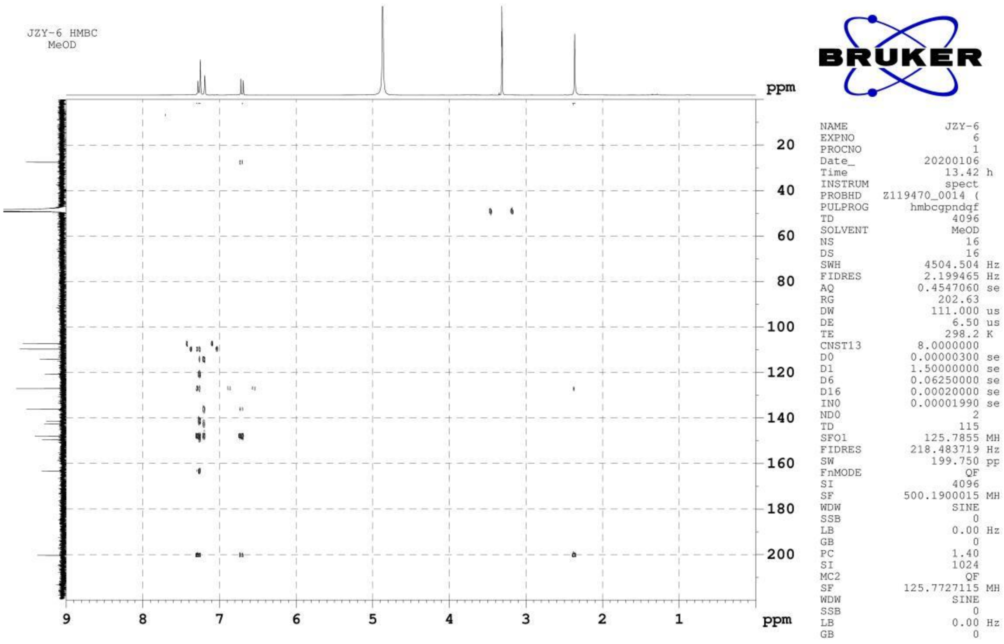

Figure S16. HMBC spectrum of compound **2.**

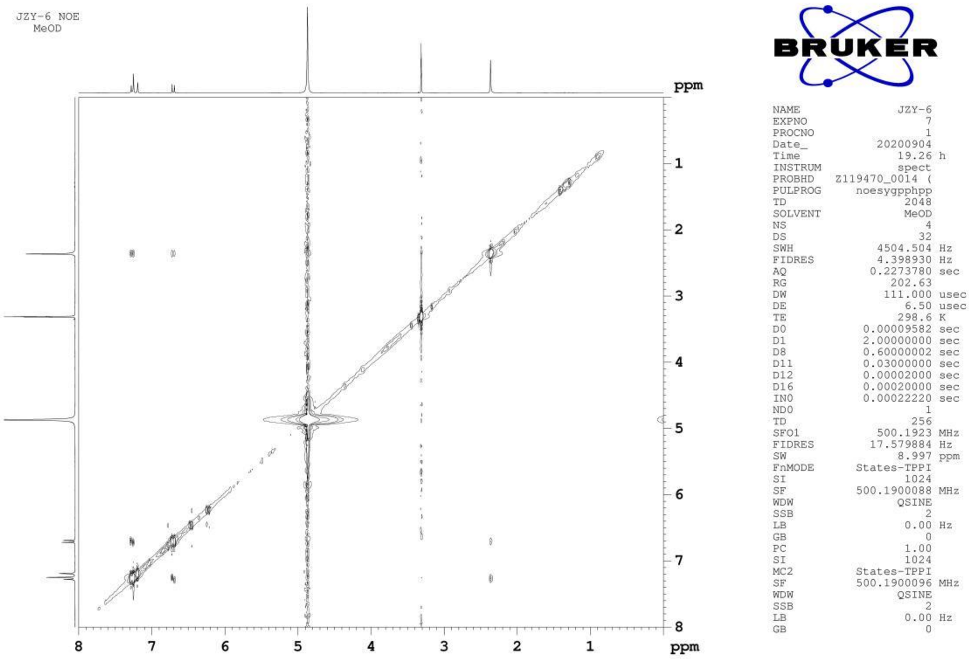

Figure S17. NOESY spectrum of compound **2**.

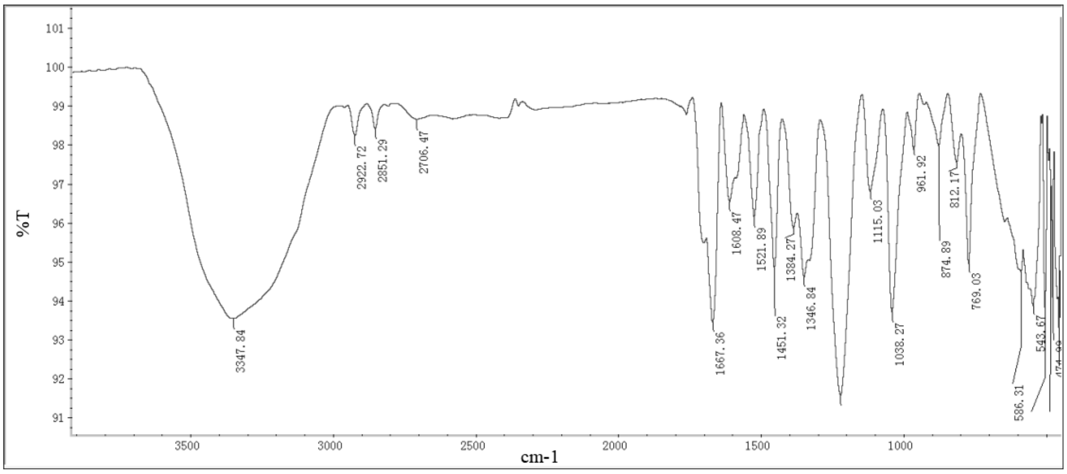

Figure S18. IR spectrum of compound **3**.

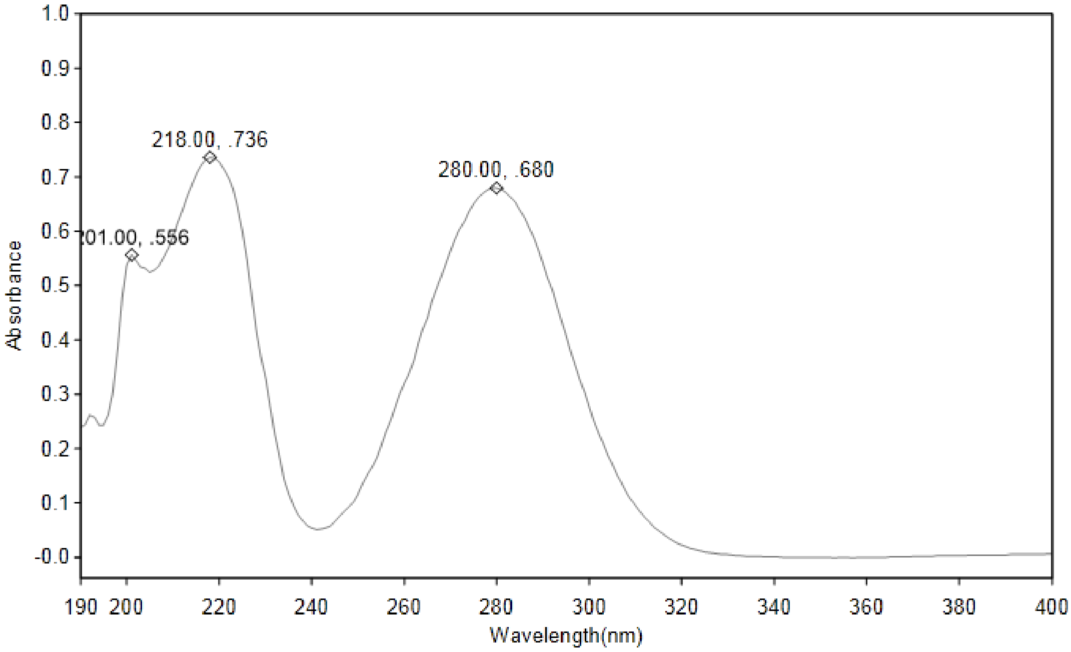

Figure S19. UV spectrum of compound **3**.

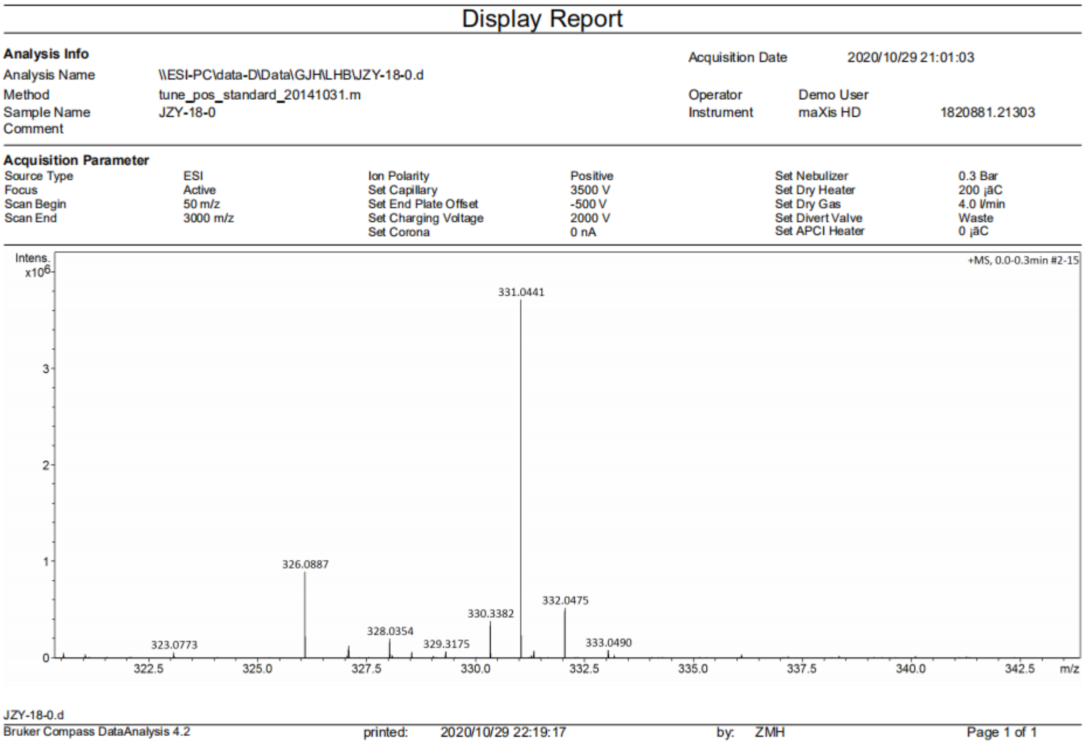

Figure S20. HRESIMS spectrum of compound **3**.

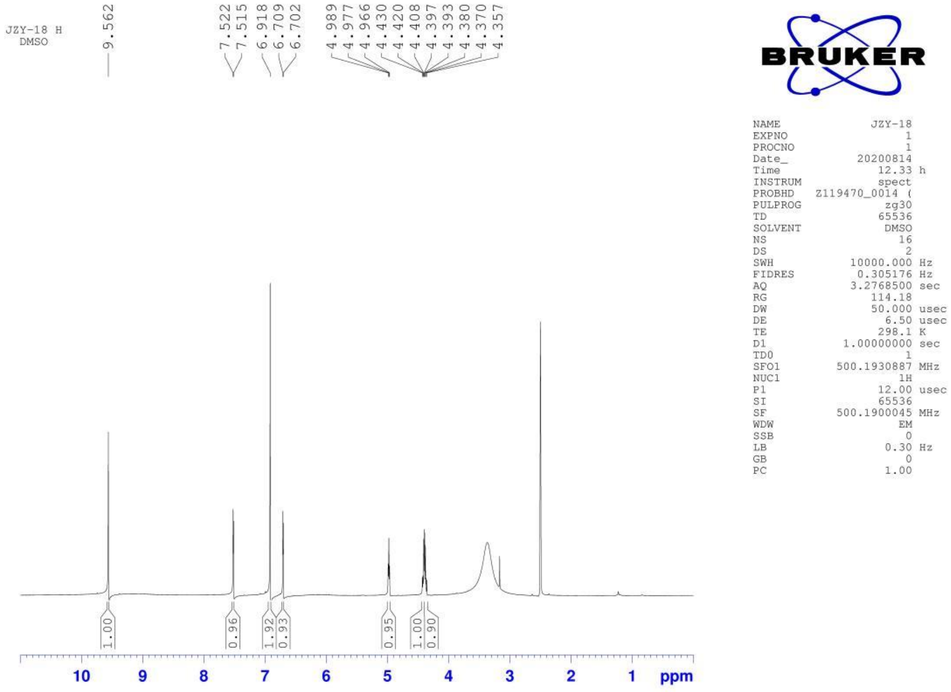

Figure S21. ^{1}H-NMR spectrum of compound **3** (MeOD, 500 MHz).

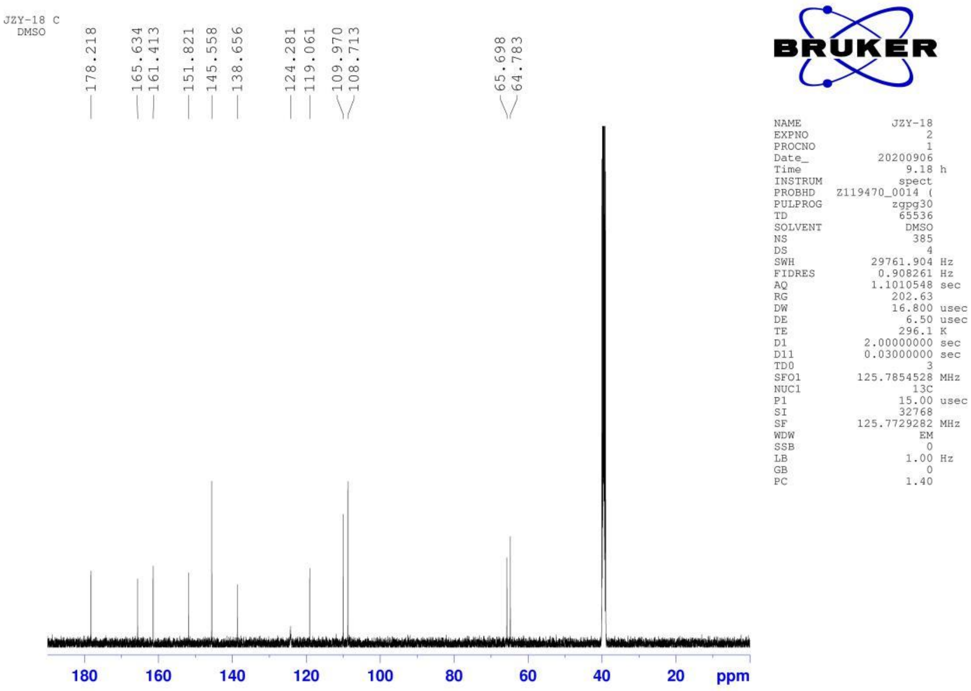

Figure S22. ^{13}C-NMR spectrum of compound **3** (MeOD, 125 MHz).

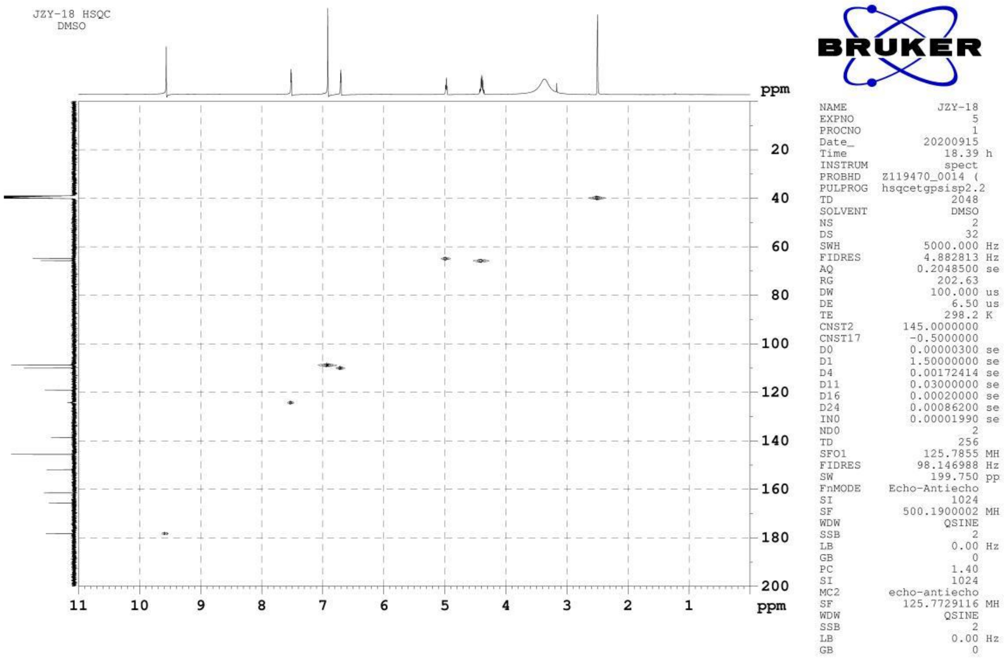

Figure S23. HSQC spectrum of compound **3**.

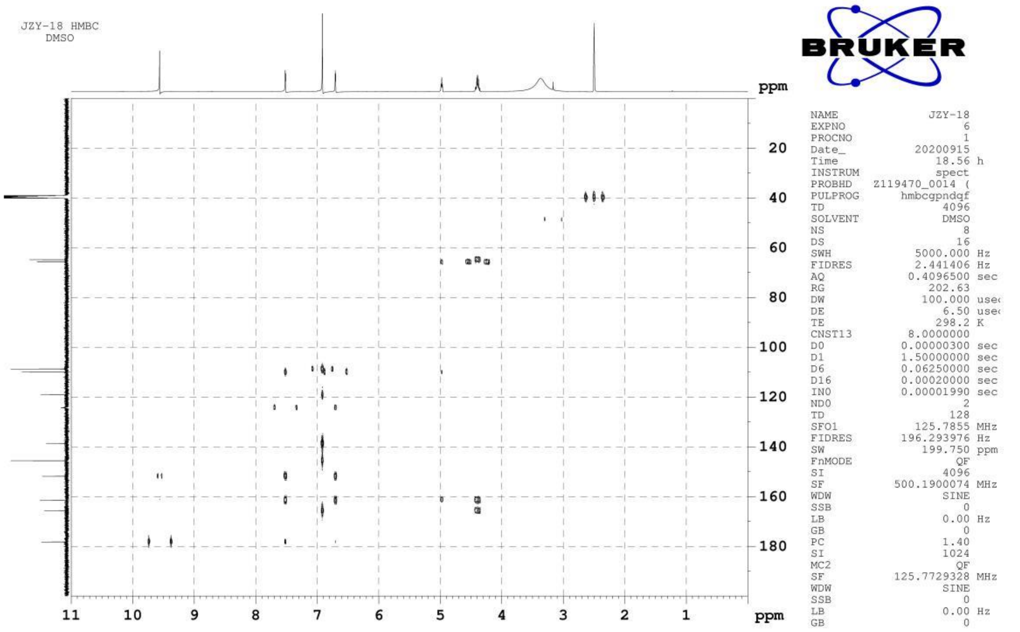

Figure S24. HMBC spectrum of compound **3**.

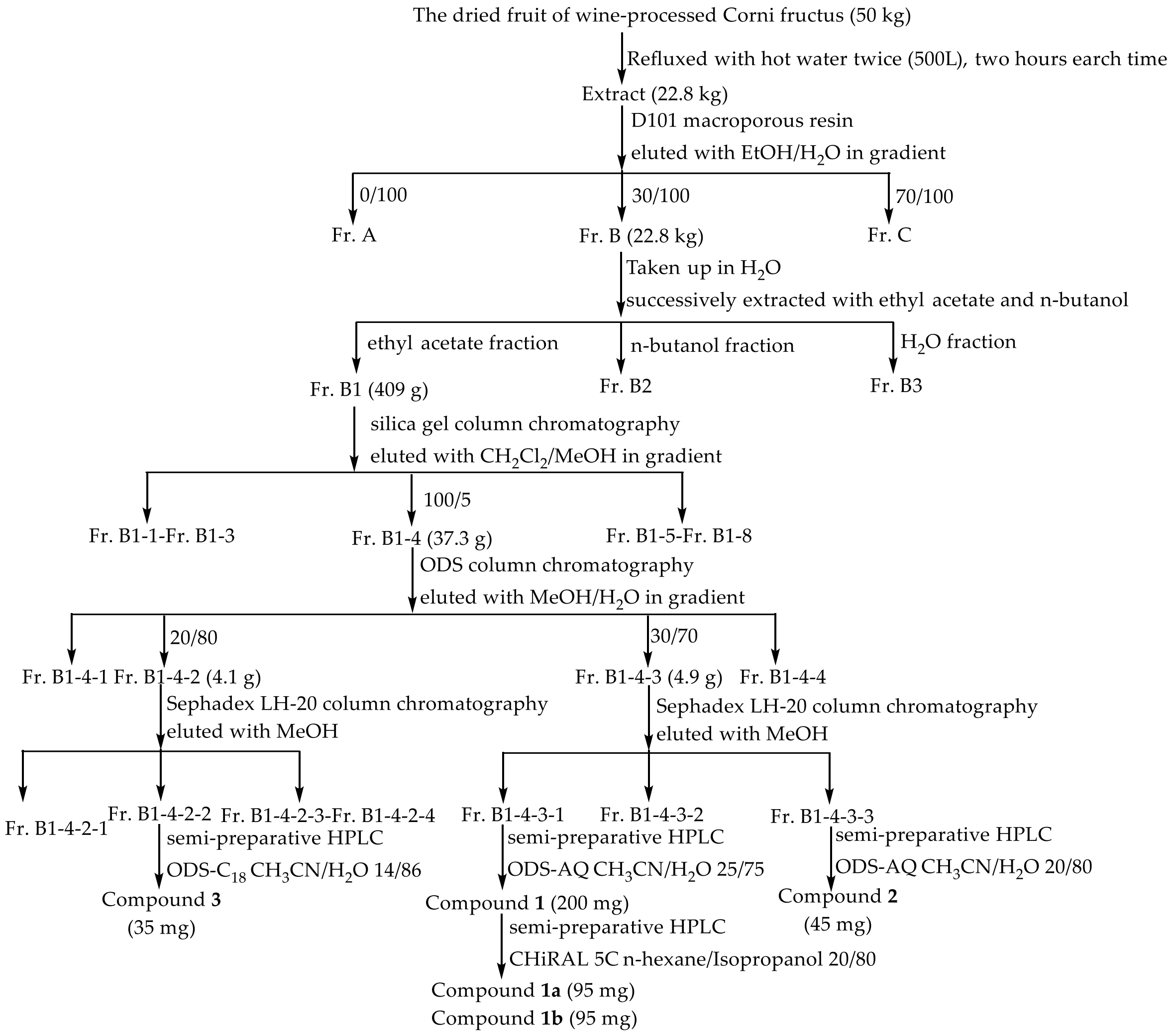

Figure S25. Separation process of compounds 1-**3**.

Table S2. Anti-inflammatory of different components on LPS-Induced RAW264.7 [a] (means ± SD,n = 3)

Sample	*c* (mg/mL)	NO inhibition rate (%)
	25	86.16 ± 5.87**
**Fr. Extract**	50	107.77 ± 3.27**
	100	113.03 ± 4.53**
	25	92.02 ± 4.91**
**Fr. 30%EtOH**	50	104.19 ± 4.37**
	100	118.94 ± 5.01**
	25	30.26 ±3 .26**
**Fr. H_2O**	50	91.36 ± 6.32**
	100	98.63 ± 4.09**
	25	46.01 ± 4.23**
**Fr. Ethyl Acetate**	50	103.33 ± 3.67**
	100	120.35 ± 5.32**
	25	45.35 ± 4.78**
**Fr. N-butanol**	50	98.03 ± 4.36**
	100	100.03 ± 3.27**
dexamethasone [b]	3	50.07 ± 2.65

[a] Values are means ± SD of three experiments, with each data point done in triplicate. [b] Dexamethasone was used as the positive control. ** $p < 0.01$.

Table S3. The cytotoxic activities of compounds **1-3** on the viability RAW264.7 cells [a] (means ± SD, n = 3)

Sample	*c* (μM)	Viability (%)
	100	125.06 ± 0.66**
	50	118.10 ± 0.32**
Compound **1**	25	119.07 ± 0.39**
	12.5	113.05 ± 0.29**
	6.25	103.12 ± 0.17**
	0	100.00
	100	168.04 ± 0.58**
	50	163.10 ± 1.06**
Compound **2**	25	134.07 ± 0.44**
	12.5	116.12 ± 0.11**
	6.25	112.09 ± 0.14**
	0	100.00
	100	118.07 ± 0.65**
	50	119.10 ± 0.43**
Compound **3**	25	127.05 ± 0.66**
	12.5	124.08 ± 1.77**
	6.25	112.03 ± 0.34**
	0	100.00

[a] Values are means ± SD of three experiments, with each data point done in triplicate.
** $p < 0.01$

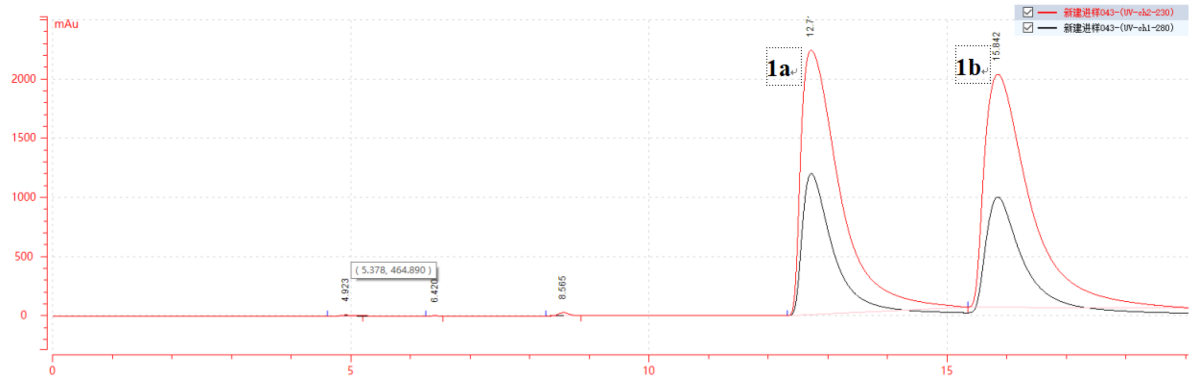

Figure S26. HPLC chart of compounds **1** chiral resolution.
